# Integrated Omics Reveal the Pathogenic Potential of *Blastocystis* sp. ST2

**DOI:** 10.1155/2024/6025236

**Published:** 2024-03-31

**Authors:** Mengjuan Cao, Shaojun Zhang, Huizhu Nan, Jing Huang, Chao Zhang, Yuxin Sun, Liwen Liu, Yuping Wang, Xin Lu, Lei Ma

**Affiliations:** ^1^Ministry of Education Key Laboratory of Molecular and Cellular Biology, College of Life Sciences, Hebei Normal University, Shijiazhuang 050024, Hebei Province, China; ^2^Hebei Collaborative Innovation Center for Eco-Environment, Shijiazhuang, Hebei Province, China; ^3^Hebei Key Laboratory of Animal Physiology, Biochemistry and Molecular Biology, College of Life Sciences, Hebei Normal University, Shijiazhuang, Hebei Province, China; ^4^Hebei Children's Hospital, Shijiazhuang 050031, China

## Abstract

*Blastocystis* sp. is a zoonotic unicellular eukaryote that is distributed worldwide. The pathogenicity of *Blastocystis* sp. has been debated over the years. In this study, mice were infected with *Blastocystis* sp. ST2 to assess the impact and underlying mechanisms on the host by integrating transcriptomics, metabolomics, and gut microbiomes. Transcriptomic analysis revealed significant differences in the expression of genes related to inflammatory cytokines, tumors, and neuropathic disease-related factors in mice infected with the parasite. A total of 430 differentially expressed genes (DEGs) were identified in *Blastocystis*-infected female mice, as compared with the control mice, and among these genes, the expression levels of 195 were upregulated (*P*  < 0.001), and that of 235 were downregulated (*P*  < 0.001). Similarly, there were different 478 DEGs in male mice, among which the expression levels of 122 genes (*P*  < 0.001) were upregulated, and that of 356 genes were downregulated (*P*  < 0.001). Kyoto encyclopedia of genes and genome analysis showed that 22 pathways in females and 28 pathways in males were enriched. Metabolomics results showed obvious metabolite changes in all mice infected with the parasite. In females, 82 different metabolites were identified, among which the expression levels of 27 metabolites were upregulated, and that of 55 metabolites were downregulated. In males, 118 metabolites were identified, among which the expression levels of 24 metabolites were upregulated, and that of 94 metabolites were downregulated. Microbiome analysis showed differences in the richness of bacterial families in *Blastocystis* sp. ST2-infected mice. LEfSe analysis showed differences in the abundance of bacterial families in female and male mice compared to the control groups. Multiomics analysis showed that the transcriptome, metabolome, and microbiome are interrelated. These results emphasize that *Blastocystis* sp. ST2 can negatively affect the host and may be a disease risk factor. The results provide insight into the mechanism of *Blastocystis* sp.–host interactions.

## 1. Introduction

*Blastocystis* sp. is a common protistan intestinal parasite found in many animals, including humans [1]. Transmission of *Blastocystis* sp. is believed to occur via animal-to-animal, human-to-human, animal-to-human, and possibly human-to-animal routes [2]. *Blastocystis* sp. has a worldwide distribution with a marked prevalence in many countries. According to most epidemiological studies, nearly all countries of the world have been classified as well-developed, with a moderate prevalence (10%–15%), or underdeveloped, with a high prevalence (55%–70%). This classification is attributed to the levels of hygiene and the presence or absence of contact with animals and contaminated water and food [3, 4]. After determining *Blastocystis* sp. in water, it has recently been listed as an important indicator for drinking water monitoring by the World Health Organization [5]. *Blastocystis* sp. is genetically diverse, and at least 40 major subtypes have been identified based on 18S rRNA. Sixteen subtypes, ST1−10, ST12, ST14, ST16, ST23, ST35, and ST41, have been reported in humans, and ST1−4 is the most common in humans, accounting for more than 90% of reports [2, 6, 7]. Due to the influence of the geographical environment and other factors, there are differences in the prevalent subtypes in different regions, and different subtypes may have differences in pathogenicity. This may also be one of the reasons why the pathogenicity of *Blastocystis* sp. has not been fully determined.

The pathogenic potential of *Blastocystis* sp. remains controversial because many epidemiological and experimental animal studies have yielded different conclusions. Previous studies have shown that *Blastocystis* sp. infection causes gastrointestinal diseases, such as irritable bowel syndrome (IBS), ulcerative colitis, mysterious chronic urticaria, chronic spontaneous urticarial, and colorectal cancer [8–13]. A significant relationship between *Blastocystis* sp. and risk factors (age, sex, education, and residence) and clinical symptoms (stomachache and nausea) has been observed, while no significant relationship has been observed between bloating and diarrhea [14]. Moe et al. [15] found accumulated inflammatory cells, swollen lamina propria, and exfoliated mucosal cells in the intestine of mice infected with the parasite. Ajjampur et al. [16] found that similar symptoms occur after *Blastocystis* sp. infection in pigs. Puthia et al. [17] reported that *Blastocystis* ratti WR1 induced host cell apoptosis and altered intestinal epithelial barriers. Surprisingly, a study provided supportive data that *Blastocystis* sp. could exacerbate existing colorectal cancer via alteration in the host immune response and increased oxidative damage [9]. Given that *Blastocystis* sp. is a gut parasite, many researchers have theorized that the parasite may alter the intestinal microbiota and have attempted to explore the effect of the parasite on the host following the above idea. Yason et al. [18] reported that ST7 *Blastocystis* sp. infection reduces the number of beneficial *Bifidobacterium* and *Lactobacillus* in the intestine, possibly leading to an imbalance in the intestinal microecology. Although the above data suggest that *Blastocystis* sp. is a pathogenic microorganism, there are reports that *Blastocystis* sp. is a normal intestinal microorganism that does not affect the body. Castañeda et al. [19] found no significant difference in the composition of the intestinal flora between *Blastocystis*-positive and *Blastocystis*-negative children. A decrease in the relative abundance of *Bacteroides* and an increase in the relative abundance of *Faecalibacterium* was observed. However, they hypothesize that the presence of *Blastocystis* is unrelated to dysbiosis at the intestinal level and that it plays a role in the ecology of the intestinal microbiota through its interactions with other microbial components [19]. Stensvold et al. [20] postulate that *Blastocystis* sp. can promote host intestinal flora balance according to the *α*-diversity of intestinal microbiota in *Blastocystis* sp. carriers, which significantly increases, while *β*-diversity shows no significant difference. Moreover, Deng et al. [21] first confirmed that *Blastocystis* sp. ST4 has a good effect on intestinal symbiotic bacteria cultured in vitro, finding that ST4 can significantly inhibit the growth of *Bacteroides vulgatus* and pathogenic *Bacillus*, speculating that ST4 may be a beneficial symbiotic subtype. In fact, *Blastocystis* sp. infection does change the normal structure of the intestinal microbiota, but whether this change is the main factor causing host discomfort is uncertain.

In recent years, apart from gut microbiota, host-expressed products and metabolites have gradually been recognized to play a key role in the health of the host. However, there is currently a lack of effective data to support the mechanism of interaction between *Blastocystis* sp. and its host. Therefore, in this study, transcriptomics, metabolomics, and intestinal microbiome analysis were combined to analyze the impact of *Blastocystis* sp. on hosts of different sex to understand the interactive mechanism of *Blastocystis* sp. and the host, determine its pathogenicity, and evaluate its public health significance.

## 2. Materials and Methods

### 2.1. Consent to Participate

Three-week-old BALB/c mice were purchased from Liaoning Changsheng Biotechnology Co., Ltd., and met ethical standards. Mice were bred and maintained in the animal facilities of Hebei Normal University providing freely accessible rodent chow and drinking water under controlled conditions. All mice were adapted for 1 week to the housing environment after purchase. When appropriate, the mice were anesthetized with an intraperitoneal injection of sodium pentobarbital (50 mg/kg), and the tested samples were quickly collected for subsequent experiments. No animals were harmed during the sampling process.

### 2.2. Parasite Culture and Preparation

*Blastocystis* sp. ST2 was identified and isolated from children's feces and preserved in the College of Life Science, Hebei Normal University [22]. The parasites were cultured anaerobically at 37°C in RPMI1640 medium containing 20% fetal bovine serum and ampicillin–streptomycin and subcultured every 2–3 days in the laboratory. After diluting the parasites, they were plated on a custom-made solid culture medium applying nutrients and ampicillin–streptomycin on the surface. Upon the growth of white colonies, they were collected and individually cultured in a medium and then obtained the parasites without impurities. *Blastocystis* sp. cysts were induced by trypsin in the medium before infecting mice.

### 2.3. Experimental Design

Twenty-four mice were divided into four groups: female control (FC) group, female test (FT) group, male control (MC) group, and male test (MT) group, with six mice in each group. The purified *Blastocystis* sp. were collected and diluted with PBS. The test group mice were orally inoculated with 1 × 10^5^ parasites, whereas the control group was inoculated with an equal dose of PBS. On the 3rd and 5th days after inoculation, feces were collected from each mouse, and DNA was extracted for PCR detection to confirm successful infection. On the 5th day, whole blood, serum, and intestinal contents were collected for omics analysis.

### 2.4. Transcriptomic Analysis

Whole blood was collected from each mouse by eyeball bleeding, and total RNA was extracted using TRIzol (Thermo Fisher Scientific, USA) according to the manufacturer's instructions. After the RNA quality control was passed, substandard cDNA libraries were obtained using AMPure XP beads using the screened mRNA. After qualified library checks, different libraries were pooled according to the amount of target on-machine data and sequenced using the Illumina HiSeq platform (Metware Biotechnology Inc.). The initial reads were obtained using Fastp, and the adapters, redundant sequences, and low-quality sequences were processed. The filtered reads were aligned with the mouse genome (Mus_musculus. GRCm39.103) using the HISAT2. Transcripts were counted using featureCounts (1.6.1), and differentially expressed genes (DEGs) among different experimental groups were analyzed using DESeq2 (1.22.2) with reference values of |log2FC| > 1, *P*  < 0.01, and false discovery rate (FDR) < 0.05. The top 10 entries (*P*  < 0.05) were selected for Kyoto encyclopedia of genes and genome (KEGG) pathway and GO functional enrichment analysis using the clusterProfiler package. Six differential genes, 1300017J02Rik, Bcl2l1, Atp5k, and Ube2s in females and Uqcr11and Birc5 in males, were selected and verified by real-time quantitative PCR (RT-qPCR), and differential gene expression was considered significant when adjusted *P*  < 0.05 by *t*-test using SPSS 20.0.

### 2.5. Metabolomic Analysis

Whole blood was collected from each mouse, and the serum was separated. The supernatants were extracted using a precooled extractant (20% acetonitrile methanol). The sample extracts were analyzed using an LC-ESI-MS/MS system (UPLC, Shim-pack UFLC SHIMADZU CBM A system, https://www.shimadzu.com/; MS, QTRAP® 6500+ System, https://sciex.com/). LIT and triple quadrupole (QQQ) scans were acquired on a triple quadrupole-linear ion trap mass spectrometer (QTRAP), QTRAP® 6500+ LC-MS/MS System, equipped with an ESI Turbo Ion-Spray interface, operating in positive and negative ion mode, and controlled by Analyst 1.6.3 (Sciex) (Metware Biotechnology Inc.). The abundance of metabolites was processed by MetaboDiff for missing values and normalized using the variance stabilizing normalization. Unsupervised PCA and supervised PLS-DA were then analyzed using ropls and mixOmics, and the variable importance point (VIP) value of each metabolite was calculated. Differential metabolites were screened according to two criteria: VIP > 1 and fold change > 2 (*P*  < 0.05). The same significantly differential metabolite, heparin, was screened out from both females and males and verified by ELISA (Mouse Heparin Sodium (HS) ELISA Kit, Shanghai Enzyme-linked Biotechnology Co., Ltd.). R (version 4.2.2) analysis was performed on the data.

### 2.6. Microbiome Analysis

Each mouse was euthanized, and the intestinal contents were removed. The total DNA of each content was extracted using the hexadecyltrimethylammonium bromide (CTAB) method. After all DNA purity and concentration, libraries for 16S rRNA gene sequencing were prepared using Phusion® High-Fidelity PCR Master Mix with GC buffer (New England Biolabs Co., Ltd.) and V3/V4 specific primers (341F : 5′-CCTAYGGGRBGCASCAG-3′, 806R: 5′-GGACTACNNGGGTATCTAAT-3′) and TruSeq® DNA PCR-Free Sample Preparation Kit (Illumina, San Diego, USA). The concentrations were quantitated by Qubit and Q-PCR, and the qualified libraries were sequenced using NovaSeq6000 (Metware Biotechnology Inc.). The original raw tags were obtained by splicing the reads using FLASH (V1.2.7, http://ccb.jhu.edu/software/FLASH/) and then filtering using QIIME (V1.9.1, http://qiime.org/scripts/split_libraries_fastq.html). Effective tags were obtained after removing chimeric sequences by blasting the species annotation database (https://github.com/torognes/vsearch/). Operational taxonomic units (OTUs) were classified on the effective tags with 97% identity using Uparse software (Uparse v7.0.1001, http://www.drive5.com/uparse/). The representative sequence of each OTU was annotated using the RDP classifier (http://rdp.cme.msu.edu/) against the Silva 16S rRNA database using a confidence threshold of 99% to obtain taxonomic classifications at the phylum, class, order, family, and genus levels. Multiple sequence alignment was performed using QIIME (V1.9.1) to further explore the phylogenetic relationships between the different OTUs. QIIME used the Shannon index to describe alpha diversity. To determine beta diversity, UniFrac distance was calculated by QIIME and visualized by principal coordinate analysis using WGCNA, STATS, and GGPLOT2 in R. Microbial functional enrichment and differential analysis were performed using PICRUSt2 (Phylogenetic Investigation of Communities by Reconstruction of Unobserved States) and microbiomeMarker in R software. Microbiome abundance and diversity between different groups were calculated using the Wilcoxon rank-sum test (Wilcoxon) and plotted in R. The linear discriminant analysis effect size (LEfSe) method was used to compare the relative abundance of all bacterial taxa between groups [23]. This method emphasizes both statistical significance and biological relevance. An LDA score of >4 and a Wilcoxon of <0.05 were used as thresholds. In enrichment analysis, the visualization of microbiome functional enrichment results was completed in R, only showing entries with *P*  < 0.05 and FDR < 0.05.

### 2.7. Joint Analysis of Multiomics Analysis

The normalized differential metabolites, genes, and microbes were subjected to Spearman's correlation coefficients. Representative products were obtained using mixOmics based on DIABLO (Data Integration Analysis for Biomarker Discovery using latent variable approaches for omics studies) modeling analysis. Strong correlations (|Spearman| > 0.5, *P*-value < 0.05) among products, metabolites, metabolites, microbes, and microbes were visualized using heat maps and Sankey diagrams. Metabolites serve as intermediaries linking the transcriptome to the microbiome. R analysis was performed on the data.

## 3. Results

### 3.1. Blastocystis sp. Causes the Differential Expression of Genes in Mice

**Figure 1:**
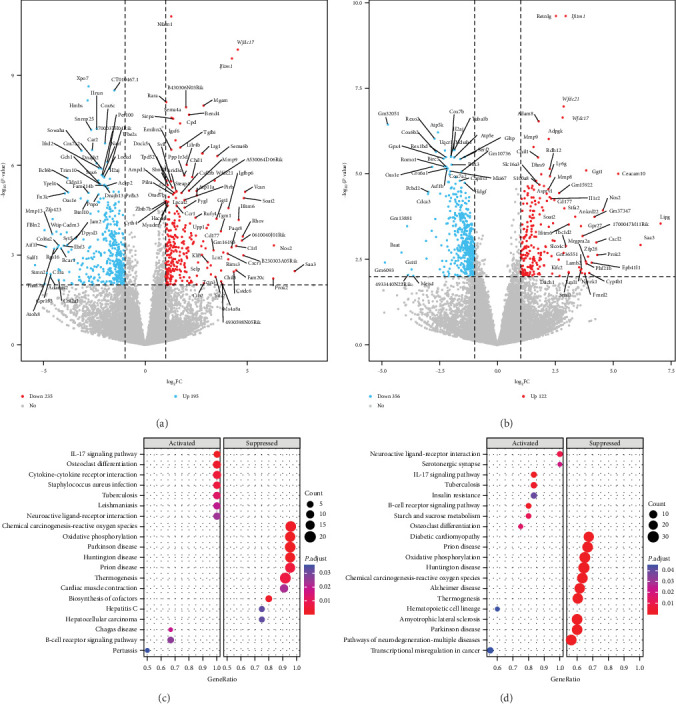
Gene differential expression analysis and KEGG analysis. (a) Differential gene volcano plot of the female FC and FT groups. (b) Differential gene volcano plot of the MC and MT groups. (c) KEGG enrichment scatter plot of the FC and FT groups. (d) KEGG enrichment scatter plot of the MC and MT groups.

Compared to the control group, the group of mice infected with *Blastocystis* sp. ST2 showed significant transcriptional differences in many genes related to inflammation, tumors, and neurological diseases. In female mice, there were 430 DEGs in the infection group (FT) compared with the control group (FC), 195 genes with upregulated expression, such as *Ifitm1, Wfdc21, Wfdc17, RARα*, and *Mgam* (*P*  < 0.001), and 235 genes with downregulated expression, such as *Xpo7* and *Hmbs* (*P*  < 0.001) (Figure 1(a)). In male mice, there were 478 DEGs in the infection group (MT) compared with the control group (MC); 122 genes with upregulated expression, such as *Ifitm1, Wfdc21, Wfdc17*, and *Adam8* (*P*  < 0.001); and 356 genes with downregulated expression, such as *Tuba1b* and *Cox7b* (*P*  < 0.001) (Figure 1(b)). The KEGG database seamlessly integrates comprehensive data on biological pathways, genomes, diseases, drugs, and chemicals and effectively amalgamates genomic information with high-level functional insights to systematically analyze voluminous data generated by high-throughput experimental techniques. KEGG enrichment analysis revealed that 22 pathways were enriched in females. The genes with upregulated expression were mainly enriched in the following pathways: osteoclast differentiation, cytokine–cytokine receptor interaction, IL-17 signaling pathway, and *Staphylococcus aureus* infection. The genes with downregulated expression were associated with oxidative phosphorylation and neurodegenerative diseases, including Parkinson's disease, Huntington's disease, and prion disease (Figure 1(c)). However, in males, 28 pathways were enriched, and genes with upregulated expression were mainly enriched in pathways such as neuroactive ligand–receptor interaction, IL-17 signaling pathway, and tuberculosis, and genes with downregulated expression had the same pathways as in females (Figure 1(d)).

**Figure 2:**
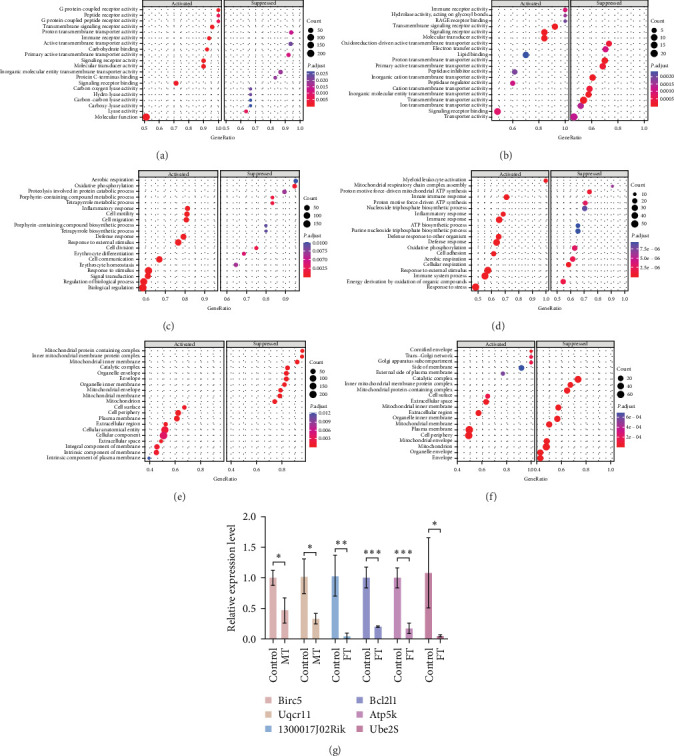
Molecular function GO enrichment scatter plots. (a) Molecular function GO enrichment scatter plots for the FC and FT groups. (b) Molecular function GO enrichment scatter plots for the MC and MT groups. (c) Biological process GO enrichment scatter plots for the FC and FT groups. (d) Biological process GO enrichment scatter plots for the MC and MT groups. (e) Cellular component GO enrichment scatter plots for the FC and FT groups. (f) Cellular component GO enrichment scatter plots for the MC and MT groups. (g) Verification results of qRT-PCR. GAPDH was the internal parameter, the data (multiple changes) were log2, expressed as mean ± SD, and the normal control group (log2 = 1) (*n* = 5) was used as the control. *⁣*^*∗*^*P*  < 0.05, *⁣*^*∗∗*^*P*  < 0.01, *⁣*^*∗∗∗*^*P*  < 0.001.

GO analysis of post-ST2 *Blastocystis* sp. infection in mice demonstrated noteworthy changes in DEGs on diverse aspects, such as molecular function, biological processes, and cellular components. After conducting a comparative analysis, it was determined that most of the genes were annotated within the context of molecular function. In the female group, unigenes were annotated to 52 molecular functions. The genes with upregulated expression were mainly annotated as G protein-coupled receptor activity, peptide receptor activity, and G protein-coupled peptide receptor activity. The genes with downregulated expression were mainly annotated to proton transmembrane transporter activity, active transmembrane transporter activity, and primary active transmembrane transporter activity (Figure 2(a)). However, in males, unigenes were annotated to 77 molecular functions, the genes with upregulated expression were in transmembrane signaling receptor activity, immune receptor activity, and molecular transducer activity, and the genes with downregulated expression were annotated to oxidoreduction-driven active transmembrane transporter activity, electron transfer activity, and proton transmembrane transporter activity (Figure 2(b)). In terms of biological processes, these genes were annotated to 292 biological processes in females. The genes with upregulated expression were annotated to inflammatory response, cell motility, and cell migration, and the genes with downregulated expression were annotated to aerobic respiration and oxidative phosphorylation (Figure 2(c)). In males, the genes were annotated to 439 biological processes; the genes with upregulated expression were mainly involved in myeloid leukocyte activation, innate immune response, and inflammatory response, and the genes with downregulated expression were mainly involved in proton motive force-driven mitochondrial ATP synthesis and mitochondrial respiratory chain complex assembly (Figure 2(d)). In terms of cellular components, in the females, the genes were in 46 cellular components, the genes with upregulated expression were mainly in the cell surface, cell periphery, and plasma membrane, and the genes with downregulated expression were mainly in the mitochondrial protein-containing complex and inner mitochondrial membrane protein complex (Figure 2(e)). In males, the genes were in 71 cellular components, and the genes with upregulated expression were mainly in the cornified envelope, trans-Golgi network, and Golgi apparatus subcompartment, while the genes with downregulated expression were mainly in the catalytic complex, inner mitochondrial membrane protein complex, and mitochondrial protein-containing complex (Figure 2(f)). From the above data, six genes with downregulated expression were screened: 1300017J02Rik, *Bcl2l1, Atp5k*, and *Ube2s* in females and *Uqcr11* and *Birc5* in males (*P*  < 0.05) and verified by RT-qPCR. These results were consistent with the transcriptomic results (Figure 2(g)), showing the reliability of the transcriptomic data.

### 3.2. Blastocystis sp. Infection Causes Metabolic abnormalities in Mice

**Figure 3:**
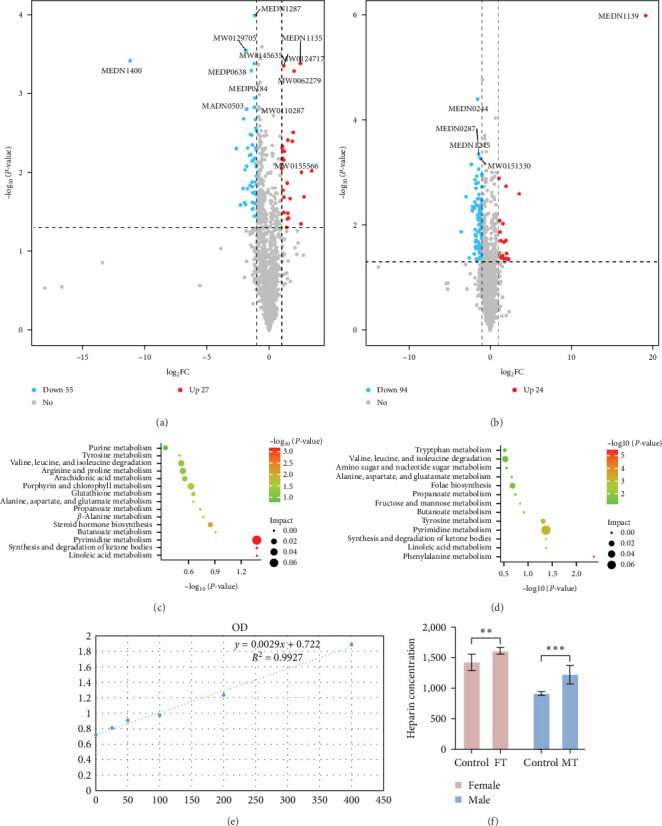
*Blastocystis* sp. infection causes metabolic abnormalities in mice. (a) Differential metabolite volcano plot of the female control group (FC) and female experimental group (FT). (b) Differential metabolite volcano plot of the male control group (MC) and the male experimental group (MT). (c) KEGG enrichment scatter plot of the female control group (FC) and female experimental group (FT). (d) KEGG enrichment scatter plot of the male control (MC) and male experimental (MT) groups. (e) Standard curve. (f) Verified results of the heparin ELISA test (*n* = 5), *⁣*^*∗∗*^*P*  < 0.01, *⁣*^*∗∗∗*^*P*  < 0.001.

To analyze the sample data space by principal components analysis, we assumed the reliability of the data and showed significant changes in metabolites between groups. The result of the volcano plot analysis displayed 82 differential metabolites in female mice and 118 differential metabolites in male mice, respectively. Twenty-seven metabolites with upregulated expression, such as MEDN1135 (heparin), MW0124717 (L-stercobilinogen), and MW0062279 (prostaglandin K2), and 55 metabolites with downregulated expression, such as MEDN1287 (LPE(0:0/16:1)), MEDN1400 (4-methoxy salicylic acid), and MW0129705 (1,7-Bis(4-hydroxyphenyl)-4-hepten-3-one), were screened in females (Figure 3(a)). Moreover, 24 metabolites with upregulated expression, such as MEDN1139 (DL-2-hydroxystearic acid), MEDN1135 (heparin), and MW0123384 (clerodin), and 94 metabolites with downregulated expression, such as MEDN0244 (orotic acid), MEDN0287 (3,5-dimethoxy-4-hydroxycinnamic acid), MEDN1245 (2-benzylmalate), and MW0151330 (Ile-Arg-Ile-Ile-Val) were screened in males (Figure 3(b)). The above differential metabolites were matched to the KEGG database to identify the pathways in which the metabolites might participate. The results of enrichment analysis showed that differential metabolites in the females were mainly enriched in pathways such as pyrimidine metabolism, synthesis and degradation of ketone bodies, and linoleic acid metabolism (Figure 3(c)) and that in males were mainly involved in phenylalanine metabolism, linoleic acid metabolism, synthesis, and degradation of ketone bodies (Figure 3(d)). Surprisingly, the level of heparin in all mice infected by *Blastocystis* sp. increased significantly and then was redetected by the enzyme-linked immunosorbent assay (ELISA). The standard curve was *y* = 0.0029 *x* + 0.722 and *R*^2^ = 0.9927 (Figure 3(e)), indicating a good fit for the experiment. Analysis of heparin concentration showed that their levels were significantly higher in the test groups than in the control groups. This was consistent with the results of metabolomics and showed the reliability of omics (Figure 3(f)).

### 3.3. Intestinal Microecology Was Changed by Blastocystis sp. in Mice

**Figure 4:**
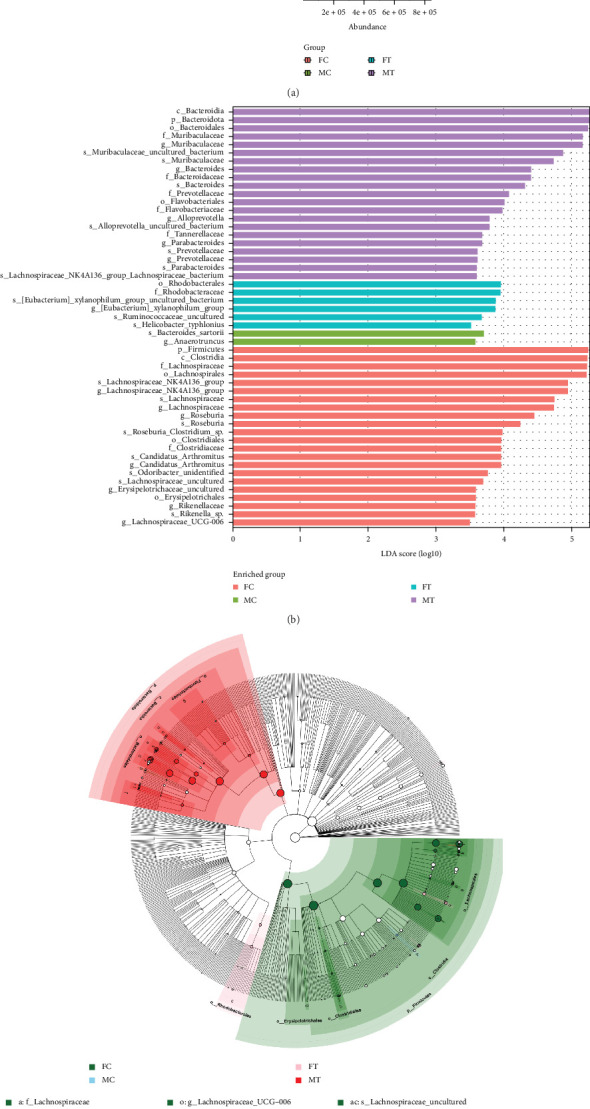
Changes in the intestinal microecology of *Blastocystis* sp. in mice: (a) gut microbiota abundance, (b) histogram of the LDA value distribution, and (c) gut microbiota evolutionary analysis.

The alteration of the intestinal flora in each group was observed based on high-throughput sequencing. Compared with the control group, the species evenness of Lachnospiraceae decreased, but that of Muribaculaceae and Bacteroidaceae increased in *Blastocystis*-infected female mice, and the same trend was observed in males (Figure 4(a)). LDA effect size (LEfSe) analysis showed a significant difference in the abundance of Rhodobacteraceae in females infected with *Blastocystis* sp. At the species level, the xylanophilum group-uncultured bacteria, Ruminococcaceae_uncultured, and *Helicobacter typhlonius* showed significant differences in abundance. In males, there were significant differences in the abundance of Muribaculaceae, Bacteroidaceae, Prevotellaceae, Flavobacteriaceae, and Tannerellaceae. At the species level, Muribaculaceae uncultured bacteria, Muribaculaceae, Bacteroides, Alloprevotella uncultured_bacteria, Prevotellaceae, Parabacteroides, and Lachnospiraceae NK4A136 groups showed significant differences in abundance (Figures 4(b) and 4(c)).

### 3.4. Joint Analysis of Transcriptomics and Metabolomics

**Figure 5:**
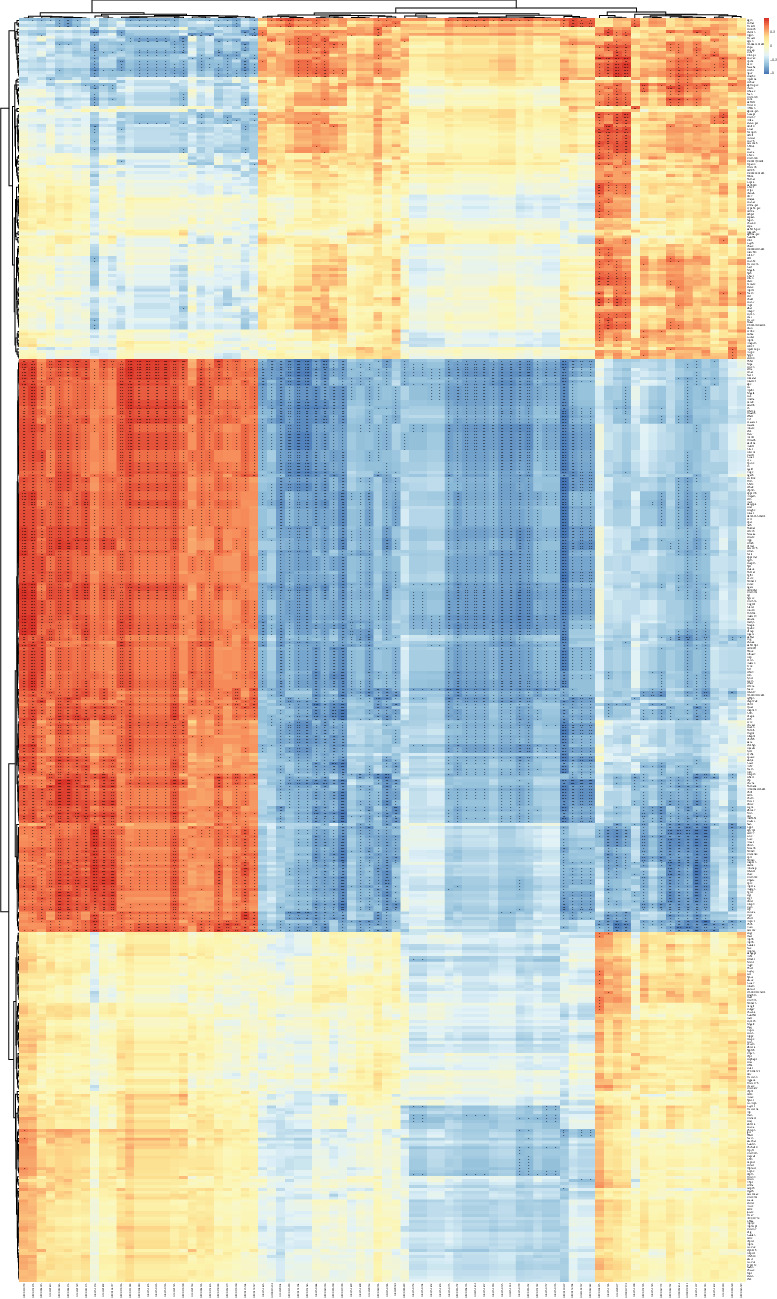
Heat map of combined transcriptome and metabolome analysis of female mice infected with *Blastocystis* sp. ST2. +0.01 < *P*  < 0.05, ++0.001 < *P*  < 0.01, +++*P*  < 0.001.

**Figure 6:**
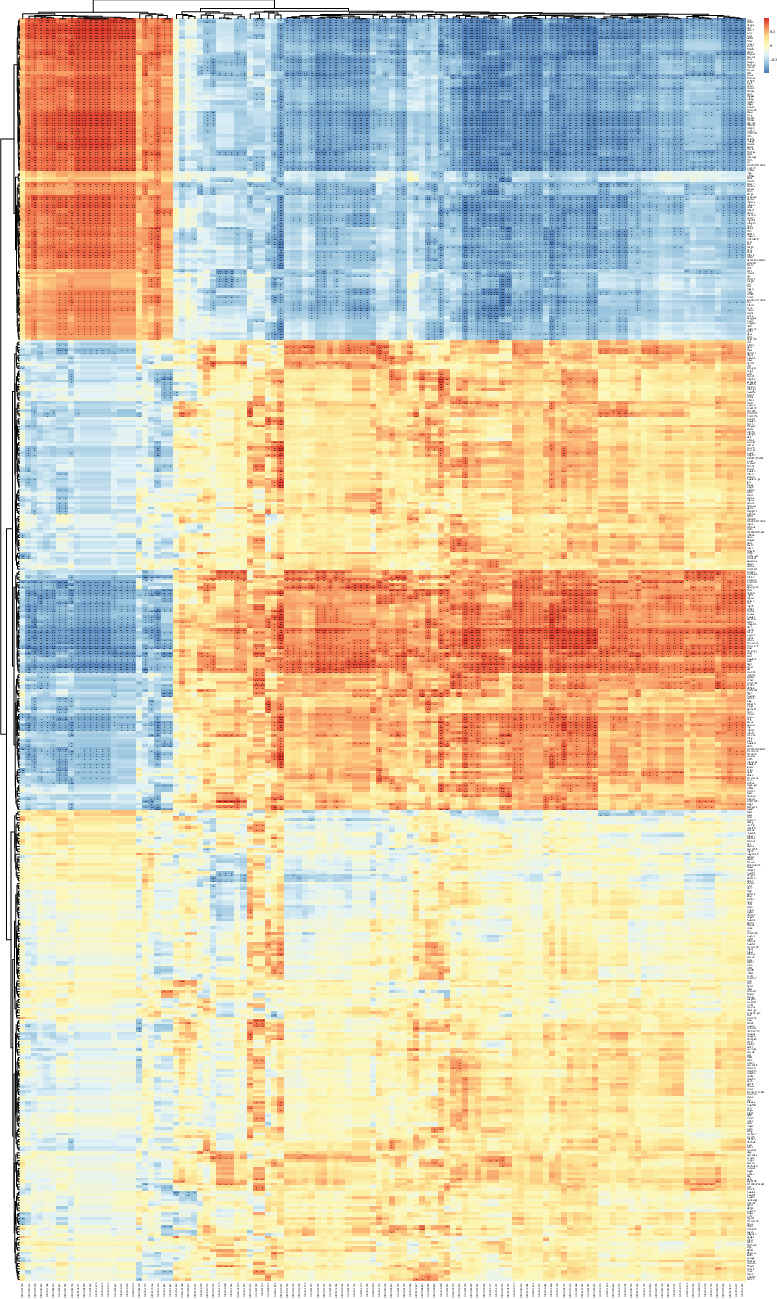
Heat map of combined transcriptome and metabolome analysis of male mice infected with *Blastocystis* sp. ST2. +0.01 < *P*  < 0.05, ++0.001 < *P*  < 0.01, +++*P*  < 0.001.

Spearman correlation coefficients were obtained by combining the transcriptomic and metabolomic methods. Heat maps were used to determine the correlation between DEGs and metabolites. In females, 329 genes correlated with 82 differential metabolites in the range of 0.01 < *P*  < 0.05, 253 genes correlated with 76 metabolites in the range of 0.001 < *P*  < 0.01, and 205 genes correlated with 50 metabolites in the range of *P*  < 0.001 (Figure 5). Similarly, in males, 366 genes correlated with 118 differential metabolites in the range of 0.01 < *P*  < 0.05, 246 genes correlated with 114 metabolites in the range of 0.001 < *P*  < 0.01, and 145 genes correlated with 74 metabolites in the range of *P*  < 0.001 (Figure 6). The association products were diverse; however, the family factors of *Ifitm* and *Wfdc* remained highlighted. In all mice, many metabolites were positively and negatively correlated with the two factors; most significantly, *Wfdc* and *Ifitm* were positively correlated with heparin and negatively correlated with LPEs. Interestingly, there were more highly significant association products in male mice than in female mice.

### 3.5. Joint Analysis of Transcriptomics and Microbiome

**Figure 7:**
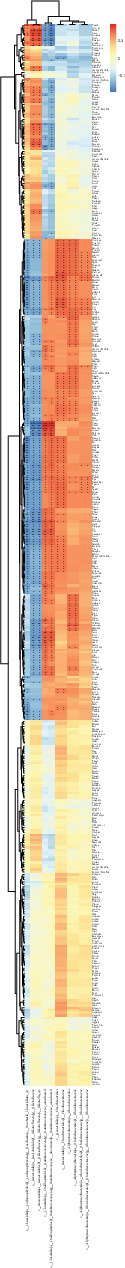
Heat map of combined transcriptome and microbiome analysis of female mice infected with *Blastocystis* sp. ST2. +0.01 < *P*  < 0.05, ++0.001 < *P*  < 0.01, +++*P*  < 0.001.

**Figure 8:**
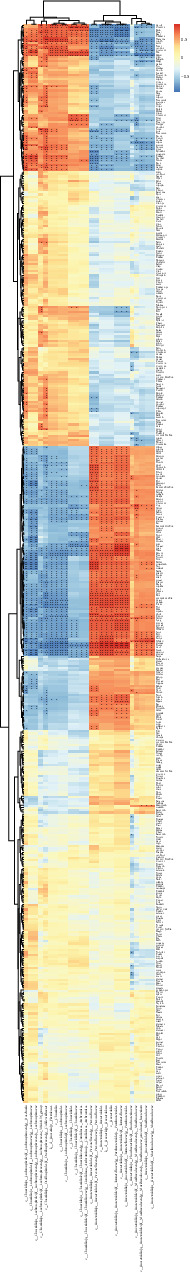
Heat map of combined transcriptome and microbiome analysis of male mice infected with *Blastocystis* sp. ST2. + 0.01 < *P*  < 0.05, ++0.001 < *P*  < 0.01, +++*P*  < 0.001.

At the class level, it was found that microbes significantly correlated with DEGs were mainly concentrated in Clostridia, Bacteroidia, and Alphaproteobacteria in the females by associating the transcriptomics with the microbiome. Clostridia was significantly negatively correlated with *Plxna4* gene (*P*  < 0.001) and significantly positively correlated with *Vcan, Chil3*, and *Fam20c* genes (*P*  < 0.001). Bacteroidia were significantly negatively correlated with *Fam20c* and *Vcan* genes (*P*  < 0.001) and significantly positively correlated with the *Hmb*s gene (*P*  < 0.001) (Figure 7). Similarly, in males, microbes that were significantly correlated with DEGs were mainly concentrated in Clostridia and Bacteroidia. Among them, Clostridia was significantly negatively correlated with the *Slc16a3* gene (*P*  < 0.001) and significantly positively correlated with *Pla2g12a* and *Gm4013* genes (*P*  < 0.001). Bacteroidia were significantly positively correlated with *Dusp1, Slc16a3*, and *Arg2* genes (*P*  < 0.001) and significantly negatively correlated with *Elob, Atp5h*, and *Mki6*7 genes (*P*  < 0.001) (Figure 8).

### 3.6. Join Analysis of Metabolomics and Microbiome

**Figure 9:**
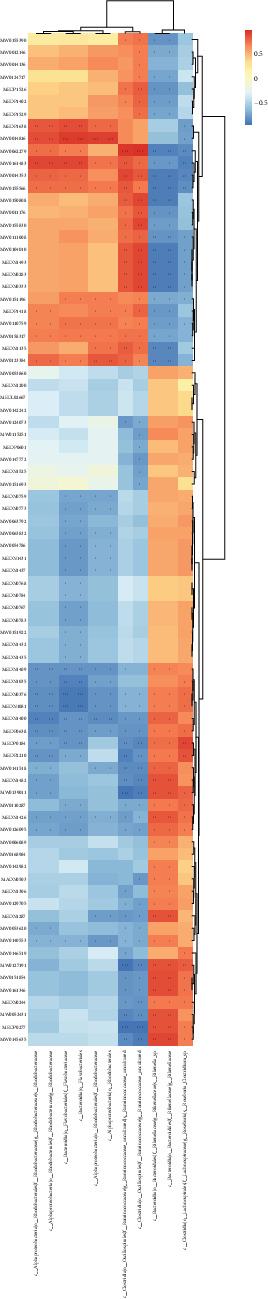
Heat map of combined analysis of metabolome and microbiome of female mice infected with *Blastocystis* sp. ST2. + 0.01 < *P*  < 0.05, ++0.001 < *P*  < 0.01, +++*P*  < 0.001.

**Figure 10:**
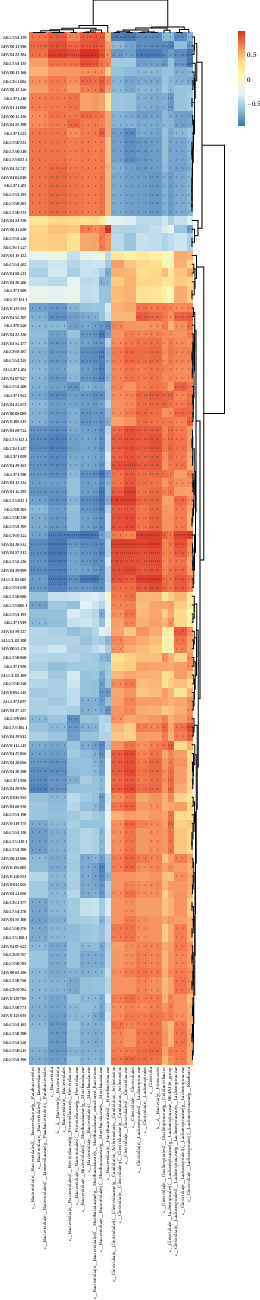
Heat map of the combined analysis of metabolome and microbiome of male mice infected with *Blastocystis* sp. ST2. +0.01 < *P*  < 0.05, ++0.001 < *P*  < 0.01, +++*P*  < 0.001.

Through a joint analysis of metabolomics and microbiome data, we aimed to understand host systematic changes in gut microbes and metabolites after *Blastocystis* sp. ST2 infection. At the class level, it was found that microbes significantly correlated with differentially expressed metabolites were mainly concentrated in Clostridia, Alphaproteobacteria, and Bacteroidia in females. Among them, Clostridia was significantly positively correlated with MW0062279 (prostaglandin K2) (*P*  < 0.001), Alphaproteobacteria and Bacteroidia were significantly positively correlated with MW0014816 (5-octynoic acid, 8-hydroxy-8-[2-(pentyloxy)phenyl]-, methyl ester) (*P*  < 0.001), Clostridia was significantly negatively correlated with MW0139011 (Mulberrofuran E), MW0127191 (1-isothiocyanatopentane) and MEDP0277 (methyl indole-3-acetate) (*P*  < 0.001), and Bacteroidia was significantly negatively correlated with MEDN0376 (9,10-DiHOME) and MEDN1081 (12,13-DiHOME) (*P*  < 0.001), respectively (Figure 9). However, in males, microbes that were significantly correlated with differentially expressed metabolites were mainly concentrated in Clostridia and Bacteroidia. Among them, Clostridia was significantly negatively correlated with MEDN1139 (DL-2-hydroxystearic acid), MW0011596 ((R)-8-Acetoxycarvotanacetone), MW0123384 (clerodin), and MEDN1135 (heparin) (*P*  < 0.001), and significantly positively correlated with MW0139362 (pinocembrin), MW0114292 (diphenol glucuronide), MEDN0121 (enterodiol), MEDN0244 (orotic acid), MW0138514 (isoquercitrin; quercetin 3-O-glucoside), MW0157312 (swertianin; 1,2,8-trihydroxy-6-methoxyxanthone), MEDN1426 (9(S),12(S),13(S)-TriHOME), and MEDL02603 (3′,4′,7-trihydroxyflavone) (*P*  < 0.001). Bacteroidia was significantly positively correlated with MW0123384 (clerodin) (*P*  < 0.001) and negatively correlated with MW0123226 (carbofuran), MEDN0287 (3,5-dimethoxy-4-hydroxycinnamic acid), MEDN1245 (2-benzylmalate), MEDP1404 (carnitine C14 : 2-OH), MW0107947 (L-pyridosine), MEDP1298 (pregnanetriol), MW0114292 (diphenol glucuronide), MEDN0121 (enterodiol), MEDP0203 (trans-Cinnamaldehyde), MEDN1426(9(S), 12(S),13(S)-TriHOME), MEDN0244 (orotic acid), MW0138514 (isoquercitrin; quercetin 3-O-glucoside), MW0157312 (swertianin; 1,2,8-trihydroxy-6-methoxyxanthone), MEDL02603 (3′,4′,7-trihydroxyflavone), and MEDN1630 (ferulic acid) (*P*  < 0.001) (Figure 10).

### 3.7. Joint Analysis of Transcriptomics, Metabolomics, and Microbiome

**Figure 11:**
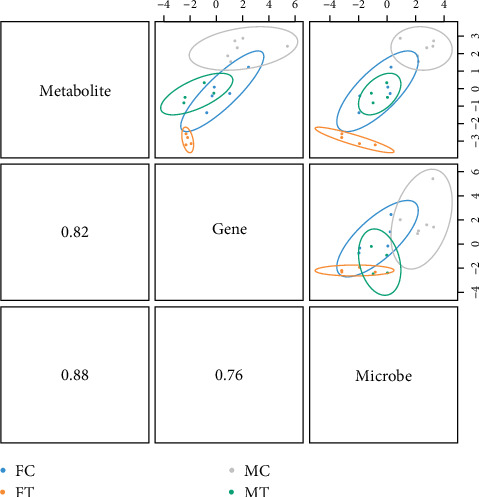
Transcriptome, metabolome, and microbiome combined analysis.

Through joint analysis of the transcriptome, metabolome, and microbiome, correlations among the three omics were exhibited (Figure 11). In females, the associated microbes were mainly concentrated in the Clostridia, Bacteroidia, and Alphaproteobacteria classes, which were associated with 301 differential genes and 128 differential metabolites (*P*  < 0.05) (*Supplementary 1*). However, the associated microbes were mainly concentrated in the Clostridia and Bacteroidia classes, associated with 307 differential genes and 158 differential metabolites in males (*P*  < 0.05) (*Supplementary 2*).

## 4. Discussion

*Blastocystis* sp. is a single-celled anaerobic eukaryotic parasite that is distributed worldwide. There are conflicting views on whether it causes disease in humans, although it has recently been linked to IBS [24]. The genetic diversity of this organism might contribute to the uncertainty regarding its role in the disease, particularly if not all subtypes have the same effect on the host. At present, there are no reports on differences in host transcriptomics and metabolomics caused by *Blastocystis* sp. Therefore, joint transcriptomic, metabolomic, and intestinal microbiome analyses were used to study the impact of *Blastocystis* sp. ST2 on the host in our study subject. Our results showed that significant changes occurred in transcription and metabolite levels and gut microbiota in mice infected with *Blastocystis* sp. ST2. However, we primarily focused on many factors in all omics associated with tissue inflammation. Previous studies have shown that *Blastocystis* sp. can cause changes in the blood, with a significant decrease in white blood cell count, hemoglobin, and hematocrit levels [25, 26]. Through the construction of a mouse model, it was confirmed that *Blastocystis* sp. caused damage to the intestinal mucosa, and pathological observation showed that the ultrastructure of the intestinal mucosal cells was significantly changed, including partial shedding of intestinal microvilli, damage to mucosal epithelial cells, local edema of mitochondria, and disordered arrangement of ridges [15, 16, 18]. The above phenomena suggest the potential harm, and we speculate that the above intestinal injuries are associated with the inflammation caused by *Blastocystis* sp. Significantly DEGs in the transcriptome are mainly related to the inflammatory response, cancer tumor occurrence, immune function, and neurological diseases. Interestingly, the transcription level of *Ifitm1* was significantly upregulated in all mice. Many studies have shown that *Ifitm1* is a marker for colorectal cancer and participates in the tumorigenesis process, and from previous studies, the overexpression of this gene could increase the probability of tumor occurrence [27]. Similarly, Kelemen et al. [28] also found that *Ifitm1*, as a negative regulator of cell proliferation, plays a key role in tumor formation according to the overexpression of the gene in tumor epithelial cells of human squamous cell carcinoma and adenocarcinoma in NSCLC patients. Coincidentally, *Blastocystis* sp. usually colonizes and damages the gut; therefore, we suspected that the parasite induces tumor formation. Moreover, parasite infection causes abnormal expression of host genes linked to the tumor. In female mice, the transcription level of *Xpo7* was significantly downregulated, and the gene is a new type of tumor suppressor that controls aging and tumor occurrence by regulating the expression of P21^CIP1^ in a previous study [29]. In male mice, upregulation in the expression of *Adam8* and downregulation in the expression of *Tuba1b* were observed. *Adam8* has been considered an important participant in invasive malignant tumors, including breast cancer, pancreatic cancer, and brain cancer, and *Tuba1b*, as a key regulator of osteosarcoma and is related to the lifetime of colon adenocarcinoma [30–32]. GO analysis revealed that after the host was infected with *Blastocystis* sp. ST2, inflammatory and immune responses occurred, which was reflected by the IL-17 signaling pathway in KEGG analysis. IL-17 is a cytokine that drives autoimmune and inflammatory diseases and promotes tumor progression in pathological environments. In addition, the Wfdc family members in all mice were matters of concern because of their functions in sperm maturation, inflammation, and innate immunity, especially *Wfdc17* and *Wfdc21* [33–37]. Interestingly, many differential factors, such as upregulated and downregulated expression of *RARα* and *Hmbs* genes, respectively, in females and downregulated expression of *Cox7b* in male mice, are similar to the host symptoms, IBS. *RARα* plays a crucial role in the selective activation of pro-inflammatory and anti-inflammatory signals, the absence of *Hmbs* can cause acute intermittent porphyria, leading to abdominal pain, neuropsychiatric disorders, and neuropathy, and *Cox7b* plays an important role in normal development of the central nervous system in vertebrates [38, 39]. Of course, it is one-sided to affirm *Blastocystis* sp. as a stimulating factor for immune diseases and tumor occurrence just by the aberrant transcripts of many genes. However, that has an important reference sense for further exploring the interaction mechanism between *Blastocystis* sp. and the host.

Changes in host metabolites caused by pathogens may cause physical illness. Many metabolites have been screened by metabonomics analysis in mice of different sex infected with *Blastocystis* sp. ST2. However, we screened and focused on several differential metabolites shared by all mice, including heparin, 4-methoxy salicylic acid, and clerodin. In a previous study, heparin had anti-inflammatory and antitumor effects [40–42], which is consistent with the effects shown by differential genes, supporting the speculation that *Blastocystis* sp. ST2 may cause pathological changes such as an inflammatory response in the host. Merida-de-Barros et al. [43] found that heparin was produced by resident white blood cells, involved in the inflammatory response, such as mast cells, and could be present at lesion sites and bind to specific receptors to release activity. Lantero et al. [44] considered heparin exhibits antimalarial activity. Thus, heparin may have an inhibitory effect on *Blastocystis* sp. and may be a protective mechanism by which the body appears to resist *Blastocystis* sp. damage. In addition to heparin, many metabolites, such as 10-hydroxydecanoic acid and 4-methylsalicylic acid, have been proven to be associated with tissue inflammation [45]. The causes of inflammation are diverse and complex, and *Blastocystis* sp. is one of the important factors based on the combined analysis of transcriptomic and metabolomic data.

Another aspect of parasite pathogenicity is expressed in the intestinal flora. However, at present, the conclusions of the study of *Blastocystis*–host interactions through the intestinal microbiome are divided. Stensvold et al. [20] and Castañeda et al. [19] hypothesized that *Blastocystis* sp. is a normal microbe in the host intestine and plays an important role in regulating intestinal flora ecology. Deng et al. [21] demonstrated that *Blastocystis* sp. ST4 could inhibit the growth of pathogenic *B. vulgatus* and suggested ST4 was a beneficial commensal. However, Nagel et al. [46] analyzed the intestinal flora of patients with IBS infected with *Blastocystis* sp. and found significant differences in the fecal microbiota between patients with diarrhea-predominant IBS and healthy controls; however, the carriage of *Blastocystis* sp. did not significantly alter the fecal microbiota. Deng et al. [47] confirmed that *Blastocystis* sp. ST2 coinfected with *Clostridium difficile* can cause life-threatening diarrhea and colitis. It is speculated that *Blastocystis* sp. ST2 may cause diarrhea in the host, but the pathogenicity of the parasite is different owing to its heterogeneity among subtypes. According to the established network relationships of the three omics, transcriptomic, metabolomic, and microbiome, Clostridia are an important microbial group that is directly or indirectly connected with transcripts and metabolites in mice infected with *Blastocystis* sp. ST2. This further indicates that *Blastocystis* sp. ST2 may damage the intestinal microecological imbalance, leading to diarrhea or pathogenicity. In this study, after *Blastocystis* sp. ST2 infection in mice, significant changes were observed in the intestinal flora, among which differences in Firmicutes and Bacteroidota were the most significant. In a previous study, the ratio of Firmicutes/Bacteroidetes (F/B ratio) was found to be a factor related to complications such as diabetes, obesity, or inflammatory bowel disease [48]. The results of this study showed that the F/B ratio decreased in mice infected with *Blastocystis* sp. ST2. This was consistent with Yañez et al.'s [48] study on *Blastocystis* sp. ST1 and ST7 and Maritat's study [49] on *Blastocystis* sp. ST4 causing changes in host intestinal microbes. Furthermore, it is speculated that IBS and weight loss may be related to the parasite. In addition, this study showed that Rhodobacterales were more abundant in the intestines in female mice infected with *Blastocystis* sp., consistent with the results of Kodio's study [50] on the changes in intestinal floras in children infected with *Blastocystis* sp.. Muribaculaceae and Prevotellaceae were two important groups of microbes in male mice infected with the parasite, consistent with the results from Kim's and Audebert's research [51, 52]. The content of other microbes, such as Lachnospiraceae, also changed with the action of *Blastocystis* sp. ST2 in mice, but their roles still require further exploration and verification. There were significant differences in gut flora between male and female mice after infection with *Blastocystis* sp. ST2, which may be related to host immunity and physiological characteristics; however, further research is required to determine whether sex effects modified the association.

In the joint analysis of transcriptomics and metabolomics, we established data relationships among different levels of molecules, combined functional analysis and metabolic pathway enrichment, and performed a correlation analysis between metabolites and microbes to achieve a comprehensive understanding of the general trend and direction of biological changes. According to the established network relationships of transcriptomic, metabolomic, and microbiome analyses, it is possible that there were some positive or negative interaction effects among the transcripts, metabolites, and microbes. However, it is very complicated in an organism and requires a lot of research data to determine how it works. The mechanism through which *Blastocystis* sp. impacts the host was studied initially, and the research data provided basis for our future research; however, complete understanding of the underlying mechanism remains elusive. We focused on inflammation, but the causes of inflammation are diverse and complex. Therefore, further in-depth research is required to determine the roles of each inflammatory factor. Additionally, the data showed subtle differences, and many studies are needed to determine whether the relationship between *Blastocystis* sp. and the host is affected by hormones.

## 5. Conclusion

Based on the analysis of transcriptomics, metabolomics, and microbiome, it is concluded that *Blastocystis* sp. ST2 infection causes significant changes in the transcript and metabolic profile of the host, as well as intestinal microecological imbalance, and it is confirmed that *Blastocystis* sp. ST2 infection is harmful to the host. These results provide insight into the mechanism of *Blastocystis*–host interactions.

## Data Availability

The data supporting the findings of this study are available from the corresponding author upon reasonable request.

## Supplementary Materials

Supplementary 1 Sankey map of the combined transcriptome, metabolome, and microbiome analysis of female mice infected with *Blastocystis* sp. ST2.

Supplementary 2 Sankey map of transcriptome, metabolome, and microbiome of male mice infected with *Blastocystis* sp. ST2.
