# Supplementary figures and images for: Integrated Omics Reveal the Pathogenic Potential of Blastocystis sp. ST2
Source: Transbound Emerg Dis. 2024 Mar 31;2024:6025236. doi: 10.1155/2024/6025236 (PMC12016975; doi:10.1155/2024/6025236)

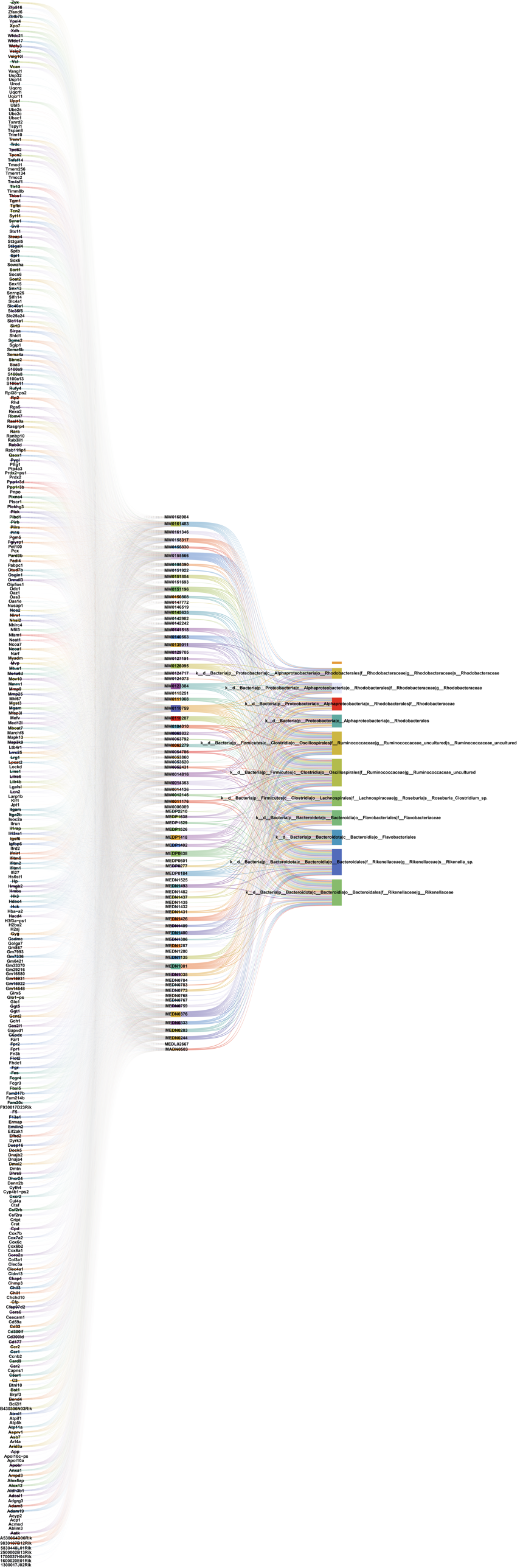

Supplement: Supplementary 1 — Sankey map of the combined transcriptome, metabolome, and microbiome analysis of female mice infected with Blastocystis sp. ST2. [file 6025236.f1.pdf]

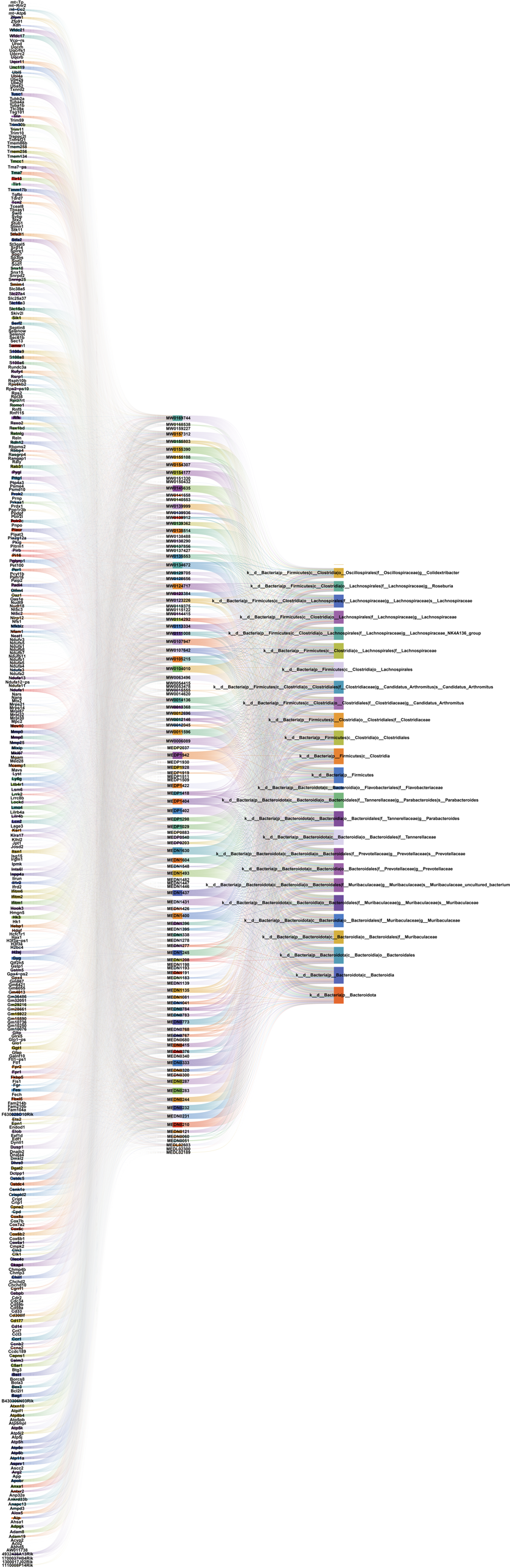

Supplement: Supplementary 2 — Sankey map of transcriptome, metabolome, and microbiome of male mice infected with Blastocystis sp. ST2. [file 6025236.f2.pdf]
